# Failure rates of mini-implants placed in the infrazygomatic region

**DOI:** 10.1186/s40510-015-0100-2

**Published:** 2015-09-15

**Authors:** Flavio Uribe, Rana Mehr, Ajay Mathur, Nandakumar Janakiraman, Veerasathpurush Allareddy

**Affiliations:** Division of Orthodontics, Department of Craniofacial Sciences, Charles Burstone Professor, University of Connecticut School of Dental Medicine, 263 Farmington Avenue, Farmington, CT 06030 USA; Private Practice, Houston, TX USA; Private Practice, Mumbai, India; Division of Orthodontics, Department of Craniofacial Sciences, University of Connecticut School of Dental Medicine, Farmington, CT USA; Department of Orthodontics, College of Dentistry, The University of Iowa, Iowa, IA USA

**Keywords:** Infrazygomatic mini-implants, Failure rate, Stability

## Abstract

**Background:**

The purpose of this pilot study was to evaluate the failure rates of mini-implants placed in the infrazygomatic region and to evaluate factors that affect their stability.

**Methods:**

A retrospective cohort study of 30 consecutive patients (55 mini-implants) who had infrazygomatic mini-implants at a University Clinic were evaluated for failure rates. Patient, mini-implant, orthodontic, surgical, and mini-implant maintenance factors were evaluated by univariate logistic regression models for association to failure rates.

**Results:**

A 21.8 % failure rate of mini-implants placed in the infazygomatic region was observed. None of the predictor variables were significantly associated with higher or lower odds for failed implants.

**Conclusions:**

Failure rates for infrazygomatic mini-implants were slightly higher than those reported in other maxilla-mandibular osseous locations. No predictor variables were found to be associated to the failure rates.

## Background

Mini-implants have become a tool to address anchorage needs in the modern orthodontic practice. They have been widely utilized for anchorage reinforcement and placed within and outside of the dentoalveolar region. Possible insertion sites for mini-implants in the maxilla include the area below the nasal spine [1], the palate [2], the alveolar process [3–5], and the infrazygomatic (IZ) crest [6]. Insertion sites other than the alveolar process allow for more versatility of orthodontic tooth movements since the roots do not interfere with tooth displacement. Specifically, the IZ crest of the maxilla is one of these anatomical sites distant from the dentoalveolar region, which allows unobstructed tooth movement, decreasing the chance of root contact.

The IZ region has important osseous characteristics such as the presence of thicker cortical bone, which allows good primary stability [7]. In fact, this region in partially edentulous patients is considered to have the best bone quality in the maxilla [8]. IZ mini-implants have been successfully used to provide skeletal anchorage for en-masse anterior retraction and intrusion of the maxillary posterior teeth [6, 9, 10].

The success and failure rate of mini-implants have been studied extensively, especially for mini-implants placed in tooth-bearing regions. Success has been defined when mini-implants are maintained in bone until the end of treatment or intentional removal, regardless of [11]. On the other hand, failure is considered as severe clinical mobility of a mini-implant that results in its inability to act as a stationary anchor, which requires removal or replacement, or loss of a mini-implant less than 8 months after placement [12–14]. Factors affecting the success and failure rate of mini-implants have been divided into different categories. These categories are patient, mini-implant, orthodontic, surgical, and mini-implant maintenance factors [11, 12, 15, 16].

There is lack of evidence in the literature specifically investigating the failure rate of mini-implants placed in the IZ region. Therefore, the objective of this study was to evaluate failure rates of mini-implants placed in the IZ crest of the maxilla and investigate the factors affecting this unfavorable outcome.

## Methods

A retrospective pilot chart review of patients that had received IZ mini-implants for orthodontic treatment from July 2007 to November 2013 was conducted at the University of Connecticut after IRB approval (IRB 13-146-3). Inclusion criteria were all patients that had received a mini-implant placed in the IZ region for use as temporary anchorage device and that had complete records. The database of the orthodontic clinic was used to search for these patients. Exclusion criteria included patients with chart notes that did not record status of the mini-implants throughout treatment. In the database search for patients that had received infrazygomatic mini-implants in our institution during the specific time period, we found a total of 40 subjects. Of these, 10 subjects were excluded from the study due to lack of complete records such as incomplete chart notes or photographs not taken at 3 months interval in the digital record.

Chart notes and photographic images of the digital charts of the patients were analyzed to evaluate the dependent and independent variables. The primary outcome was mini-implant failure. Independent variables associated to mini-implant success were as follows: patient-, mini-implant-, orthodontic-, surgical-, and mini-implant maintenance-related factors. These independent variables were evaluated as predictors of mini-implant failure.

**Fig. 1:**
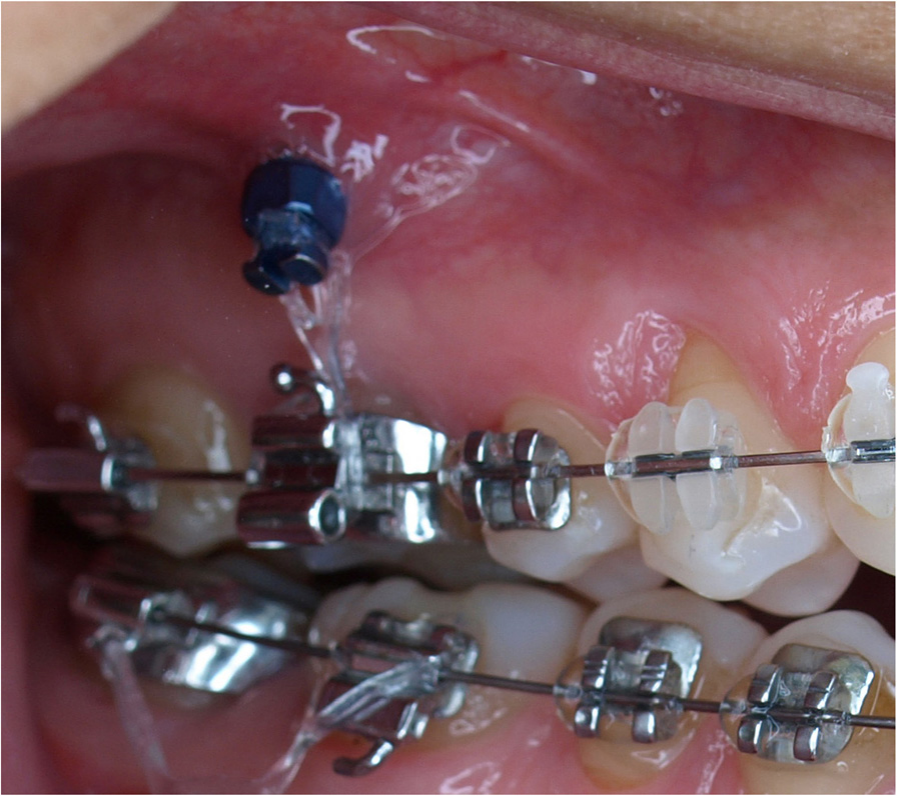
Mini-implant placed in the IZ region of the maxilla

Data from a total of 30 consecutive patients (mean age 22.2 ± 11 years) who had 55 IZ mini-implants placed and met the inclusion criteria was collected (Table 1). Four different types of mini-implants [Lomas (Mondeal, Tuttligen, Germany), Imtec (Unitek 3M, Monrovia, California), Aarhus (Medicon, Tuttligen, Germany), Dual Top (RMO, Denver, Colorado)] were used and analyzed. The brand selection for each patient was based on the availability of the mini-implant system and mini-implant in the clinic at the time of placement. The surgical procedure included local anesthesia followed by a small tissue punch. A pilot hole was placed with a manual driver for 22 of the 55 mini-implants. The same manual driver was used for the pilot hole regardless of the brand of the mini-implant. Placement of the mini-implants was performed with the specific driver designed for that particular system by the manufacturer. The mini-implants were placed by two types of operators, an experienced clinician (more than 50 mini-implants placed, FU) and by residents under direct supervision of this operator. Nine orthodontic residents placed the mini-implants during this time period, all of whom had minimal experience in mini-implant placement (less than 10 mini-implants placed). All mini-implants were placed at an approximate angle of 40° to 70° to maxillary occlusal plane in the IZ area by palpating the “key ridge” above the first permanent molar (Fig. 1) [7, 10].

**Table 1:** Failure rates by characteristics of patients

Characteristic		Total *N*	Failure rate (%)
Age	<18 years	29	20.69
	≥18 years	26	23.08
Gender	Male	13	38.46
	Female	42	16.67
Presence of medical condition	Yes	6	50
	No	49	18.37
Lomas (Mondeal, Tuttligen, Germany)	Yes	43	18.60
	No	12	33.33
Diameter of implant	1.50 or 1.80	10	30
	2.00 or 2.30	45	20
Length of implant	6 to 8 mm	13	30.77
	9 mm	42	19.05
Force magnitude	150 g	12	16.67
	>150 g	43	23.26
Type of movement	Purely intrusive	25	16
	All others	30	26.67
Oral hygiene	Poor	14	35.71
	Good or fair	41	17.07
Operator experience	Experienced	11	18.18
	Inexperienced	44	22.73
Use of pilot hole	Yes	22	13.64
	No	33	27.27
Side of implant	Left	28	25
	Right	27	18.52

After placement, initial stability of the mini-implant was checked by the operator who ensured there were no signs of mobility. The mini-implants were used for the retraction, distalization, and intrusion purposes. Combination of these movements such as intrusion–retraction and intrusion–distalization was also recorded. The mini-implants were loaded with these types of forces for an average of 13.67 ± 6.79 months. The primary outcome variable of interest was implant failure of the mini-implants.

Failure was defined as a mini-implant that had to be removed or had fallen out after placement. The effect of oral hygiene on survival of the mini-implants was assessed. The sample was divided into three groups depending on the patients’ oral hygiene: good, fair, or poor, and was determined based on the photographic images and notes from the record. Vertical facial pattern was evaluated based on the mandibular plane angle (MPA) and Frankfurt/mandibular plane angle (FMA).

### Statistical analysis

The outcome of interest was “failure” of implants. This was modeled as a binomial variable (Failure—yes/no). Simple descriptive statistics were used to summarize the estimates of failure rates across different levels of predictor variables. The predictor variables examined included age, gender, presence of medical conditions, mini-implant brand (Lomas versus others), diameter of implant, length of implant, amount of force used, type of movement, oral hygiene, operator experience, use of pilot hole, and side of infrazygomatic implant. Univariate logistic regression analyses were used to examine the independent association of each predictor variable with the outcome (failure of implants). The regression models were fit using Generalized Estimating Equations method. The effect of clustering of outcomes within patients was adjusted in the models. An exchangeable correlation matrix was specified. Odds ratio for each characteristic that resulted in a failed mini-implant was calculated. Each individual implant was the unit of analysis. All tests were two sided and a *p* value of <0.05 was deemed to be statistically significant. All statistical analyses were conducted using SPSS Version 22.0 (IBM Corp, New York City, NY) and SAS Version 9.3 (SAS Institute, Cary, NC).

## Results

Table 1 describes all the patient-, implant-, orthodontic-, surgical-, and implant maintenance-related characteristics of the patient sample and mini-implants placed. Of the 30 patients (55 mini-implants) that received IZ mini-implants, majority were female, with no significant medical condition. Approximately 80 % of the mini-implants were Lomas (Mondeal, Tuttligen, Germany) with a 2 × 9 mm dimension. All mini-implants were placed in unattached gingiva almost evenly distributed between left and right sides and immediately loaded. Most patients maintained good or fair oral hygiene and no infection developed around any of the mini-implants. A loading force of 200 g for intrusion of posterior teeth purposes was used on the majority of mini-implants. The average MPA and FMA angles were 39.9° ± 6.60° and 31.3° ± 6.36°, respectively.

Over the course of treatment, 21.8° % of the mini-implants failed. Overall, failure rates were higher among those aged ≥18 years, males, with medical conditions, use of non-Lomas implants, implants with length of 6 to 8 mm (compared to 9 mm), implants with 1.5/1.8 mm diameter (compared to 2 or 2.3 mm diameter), use of force greater than 150 g, with poor oral hygiene, when placed by inexperienced operators, and left-sided implants. Purely intrusive movements had lower failure rates when compared to all other combinations of movements. Failure rates were lower when pilot holes were used. The results of the univariate logistic regression analyses are summarized in Table 2. The estimates from the regression models indicated that none of the predictor variables were significantly associated with higher or lower odds for failed implants.

**Table 2:** Estimates from univariate regression analysis (independent association between predictor variables and failed implants)

Predictor variable		Estimate	Odds ratio	*p* value
Age	<18 years	−0.1182	0.89	0.86
	≥18 years	Reference		
Gender	Female	−1.1383	0.32	0.11
	Male	Reference		
Presence of medical condition	Yes	1.50	4.48	0.14
	No	Reference		
Lomas (Mondeal, Tuttligen, Germany)	Yes	−0.7983	0.45	0.31
	No	Reference		
Diameter of implant	1.50 or 1.80	0.5630	1.76	0.40
	2.00 or 2.30	Reference		
Length of implant	6 to 8 mm	0.7078	2.03	0.37
	9 mm	Reference		
Force	150 g	−0.4282	0.65	0.59
	>150 g	Reference		
Type of movement	Purely intrusive	−0.6377	0.53	0.34
	All others	Reference		
Oral hygiene	Poor	1.0269	2.79	0.09
	Good	Reference		
Operator experience	Experienced	−0.3145	0.73	0.6848
	Inexperienced	Reference		
Use of pilot hole	Yes	−0.8610	0.42	0.32
	No	Reference		
Side of implant	Left	0.3962	1.49	0.53
	Right	Reference		

## Discussion

A recent meta-analysis reported that the average overall success rate of mini-implants to be approximately 86 % [14]. This analysis included studies for mini-implants placed in different maxillomandibular locations. However, the vast majority of the studies reporting on mini-implant failure rate have predominantly focused on those placed in interradicular sites [3, 4, 11, 17, 18]. The findings of our study show that IZ mini-implants have slightly lower success rate (78.2 %) than that of the average mini-implant. This is in contrast to Liou et al.’s [6] findings who reported 100 % success of mini-implants placed in this region.

The reason for the different results in success rates with our study may be attributed to the size of the mini-implants. In their study, the length of the mini-implants was 17 mm. Additionally, the success rate on that study was based on a limited time period of 9 months compared to our study where mini-implants were loaded for an average of 13 months. Furthermore, mini-implant mobility, recorded as displacement, was reported in Liou’s study in 44 % of the patients. Thus, failure could have been evidenced at a later time point for these patients. Finally, although the mini-implants in our study were either placed by an experienced operator, or supervised by an experienced operator who had placed more than 50 mini-implants, our experience in placement of the IZ mini-implants developed through the duration of the study. It is possible that the perfect success rate reported in Liou’s study might be related to experienced operators with more than 50 mini-implants placed in this specific region.

One important variable for the different success rates of mini-implants is skeletal facial pattern. Moon et al. [19] found similar success rates (77 %) to those of our study for mini-implants placed interdentally in patients with high Frankfurt-mandibular plane angle (FMA). This skeletal type was prevalent in the majority of our patients where the average FMA and mandibular plane angle (MPA) was 31.3° and 39.9°, respectively. This finding is also in agreement with a study by Miyawaki et al. [3] who also reported that mini-implants placed in patients with high MPA had lower success rates (72.7 %). Indeed, it has been found that patients with an increased vertical skeletal pattern have reduced cortical bone thickness, which may affect primary stability of the mini-implants [20]. However, it is unknown if this reduced cortical bone thickness is also present in the infrazygomatic region.

An evaluation of patient-, mini-implant-, orthodontic-, surgical-, and mini-implant maintenance-related factors that could affect the stability of mini-implant was performed. Among all these factors, none were associated with greater odds of failure. Poor oral hygiene showed a trend to be associated to failure rates. Although this is an expected finding, there is controversy of the role of oral hygiene in mini-implant failure. Sharma et al. [21] reported that poor oral hygiene and inflammation were associated to mini-implant failure. On the other hand, Park et al. [11] found that oral hygiene played no role, but local inflammation around the mini-implants did.

Perhaps the type of mucosa surrounding the mini-implant may play a more important role in the inflammatory reaction and thus the success of the mini-implant. It has been reported that nonkeratanized gingiva may be a risk factor for mini-implant failure. Viwattanatipa et al. [22] found low survival rates of mini-implants placed in the infrazygomatic region or vestibular area (46 % after 1 year). In this study, all the mini-implants were placed on nonkeratanized tissue which could be less resistant to the effects of plaque and thus compromise mini-implant stability. Possibly a longer mini-implant that approximates the attached gingiva may reduce the potential for the development of an inflammatory process.

Although there were some mini-implants that became mobile, some of these did not fail. This is consistent with the findings of Liou et al. [6] who specifically evaluated IZ mini-implants and found this type of screws have some degree of mobility without failure. However, we observed that mobility appeared to be closely related to failure.

One surprising finding was the fact that operator experience was unrelated to mini-implant failure. Since this type of mini-implant placement has more technical difficulty, it was expected that non-experienced operators would have more failures. This nonsignificant finding in the regression analysis may be related to the fact that these mini-implants, although placed by residents, were still supervised by the experienced operator.

One important factor that could contribute to the failure rate is the angle of placement and the direction of loading force in mini-implants placed in the IZ region. In fact, Perillo et al. [23] found in a recent study using a finite element analysis that the insertion angle of the mini-implant and the direction of force have a significant influence in the stress on the bone. This parameter was not evaluated in the present study, as it would have needed to be examined in a prospective nature. Moreover, recording the direction of the force vector may be difficult as it may vary as treatment progresses based on the biomechanical needs.

The main limitation of this study is its retrospective nature. Although success rates can be reported when a categorical variable is reported as yes or no, the factors associated to these failures are more difficult to extract from chart notes. In fact, the selected patients from the clinic database may have not accounted patients where the IZ mini-implant was placed and removed immediately due to inadequate primary stability, thus underestimating the true failure rate. Although there is a possibility for this, based on the authors’ experience, inadequate primary stability of the mini-implants in this IZ region has rarely been observed. Regardless of these limitations, this study provided data for expected success rates in mini-implants placed in the IZ region, which from a biomechanical perspective, provide significant versatility for orthodontic tooth movements difficult to achieve from anchorage drawn from mini-implants placed in interradicular sites.

The present study was designed to be a pilot explorative study of a multitude of patient- and provider-related factors associated with failure of IZ mini-implants. A multitude of patient- and provider-related factors could influence outcomes (in this case—failure of IZ mini-implants) and the precise role of each variable on the outcome is difficult to elucidate with a small sample size. This is particularly true when there are variations in the distribution of covariates. The current study was designed to be a pilot project and we identified a mix of patient-related factors that are associated with IZ mini-implant failures. We intend to use results from the present study to design a future prospective study to identify factors associated with failure of IZ mini-implants. Our study included 30 patients (55 mini-implants). These patients were selected based on a chart review over a 6-year time period. Our unit of analysis was each individual mini-implant. In effect, our sample size was 55. This number is still inadequate and the present study may be underpowered considering the number of variables we included in the regression models. It is difficult to increase the sample sizes for such single center studies owing to the fact that very few patients elect to have IZ mini-implants and relatively few number of orthodontists place the IZ mini-implants. The solution will be to increase sample sizes by conducting multi-center studies where we can capture an adequate number of patients that are also heterogeneous in terms of covariate distribution. The present study results will aid in designing better controlled multi-center prospective studies. Therein lies the importance of the present study.

## Conclusions

- Mini-implants placed in the IZ region had a 21.8 % failure rate. This failure rate is slightly higher than that reported for mini-implants placed interradicularly.
- Patient, mini-implant, orthodontic, surgical, and mini-implant maintenance factors were not predictive of failure rates.
